# Association between three functional microRNA polymorphisms (miR-499 rs3746444, miR-196a rs11614913 and miR-146a rs2910164) and breast cancer risk: a meta-analysis

**DOI:** 10.18632/oncotarget.13426

**Published:** 2016-11-17

**Authors:** Hong Zhang, Yafei Zhang, Wanjun Yan, Wen Wang, Xixi Zhao, Xingcong Ma, Xiaoyan Gao, Shuqun Zhang

**Affiliations:** ^1^ Department of Oncology, Second Affiliated Hospital, School of Medicine, Xi'an Jiaotong University, Xi'an, Shaanxi, China; ^2^ Department of General Surgery, Second Affiliated Hospital, School of Medicine, Xi'an Jiaotong University, Xi'an, Shaanxi, China

**Keywords:** breast cancer, rs3746444, rs11614913, rs2910164, meta-analysis

## Abstract

Three functional microRNA polymorphisms (miR-499 rs3746444 A > G, miR-196a rs11614913 C > T and miR-146a rs2910164 G > C) have been reported to be associated with breast cancer (BC) risk. However, the results of the published studies are inconsistent. In order to obtain a more credible result, we conducted this meta-analysis. We searched PubMed, EMBASE and Web of Science databases to identify relevant studies. Pooled odds ratios (ORs) and 95% confidence intervals (CIs) were used to assess the association. Thirty-eight eligible studies with 17,417 cases and 18,988 controls were included in this meta-analysis. Our results showed that the rs3746444 was associated with an increased breast cancer risk in the four genetic models (G vs. A: OR = 1.17, *P* = 0.008; GG vs. AA: OR = 1.41, *P* < 0.001; AG vs. AA: OR = 1.10, *P* = 0.036; GG+AG vs. AA: OR = 1.16, *P* = 0.001). In the subgroup analysis by ethnicity, significant correlation remained in Asians but not in Caucasians. For rs11614913, obvious decreased breast cancer risk was observed in Caucasian populations (T vs. C: OR = 0.93, *P* = 0.044). However, we couldn't detect an association between rs2910164 and breast cancer risk. This meta-analysis demonstrates that rs3746444 could increase breast cancer risk in Asians and in general populations, while rs11614913 could decrease the risk of breast cancer in Caucasians. The rs2910164 polymorphism has no association with breast cancer risk. More multicenter studies with larger sample sizes are required to verify our results.

## INTRODUCTION

Breast cancer is the most common malignancy tumor among women, which accounts for 25% of all cancer cases in women all over the world, and it is the principal cause of female cancer-related death [1]. In the United States alone, a total of more than 2.8 million women suffered from breast cancer in 2015, and the morbidity of breast cancer is still increasing fast in recent years, so breast cancer has become a serious threat to the health and life of women worldwide [2]. The occurrence and development of breast cancer is a multistep, multistage complicated process involving multiple factors, among which genetic factors are considered to play a crucial role [3]. Consequently, identifying susceptible gene of breast cancer is of great importance, which can lead to better diagnosis, treatment and possible prevention of breast cancer.

MicroRNAs (miRNAs) are a class of non-coding single-stranded RNA molecules of about twenty-two nucleotides encoded by endogenous genes. By binding to the complementary sequence of the 3′ untranslated region of the specific target gene mRNA, microRNAs can degrade mRNA or inhibit its translation, and thus regulate the expression of target gene [4]. MiRNAs are highly conserved, tissue-specific and taking part in the regulation of many physiological and pathological process, such as cell differentiation, cell proliferation, cell apoptosis, fat metabolism, etc [5, 6]. MiRNAs have many important functions: among them, the function of microRNAs in cancer occurrence and progression attracts the most attention [7].

Studies have reported that single nucleotide polymorphisms (SNPs) or genetic mutations occurring in miRNAs could affect the efficiency of miRNA binding to the target sites of mRNA and alter the expression of related gene, which may involve acquisition of cancer susceptibility, so miRNAs play an important role in initiation and development of malignancies [8]. So far there are many studies about microRNA polymorphisms (miR-499 rs3746444, miR-196a rs11614913 and miR-146a rs2910164) and breast cancer susceptibility, but the results are controversial [9–26]. In addition, two meta-analyses on this issue published in 2013 and 2015 yielded inconsistent results: one reported that rs3746444 and rs2910164 were not associated with breast cancer risk [27], while another showed that these two microRNA polymorphisms could increase breast cancer susceptibility [28]. Therefore, we conducted this meta-analysis including some latest studies to make a more accurate and comprehensive assessment of these three polymorphisms and breast cancer risk.

## RESULTS

### Characteristics of included studies

**Figure 1:**
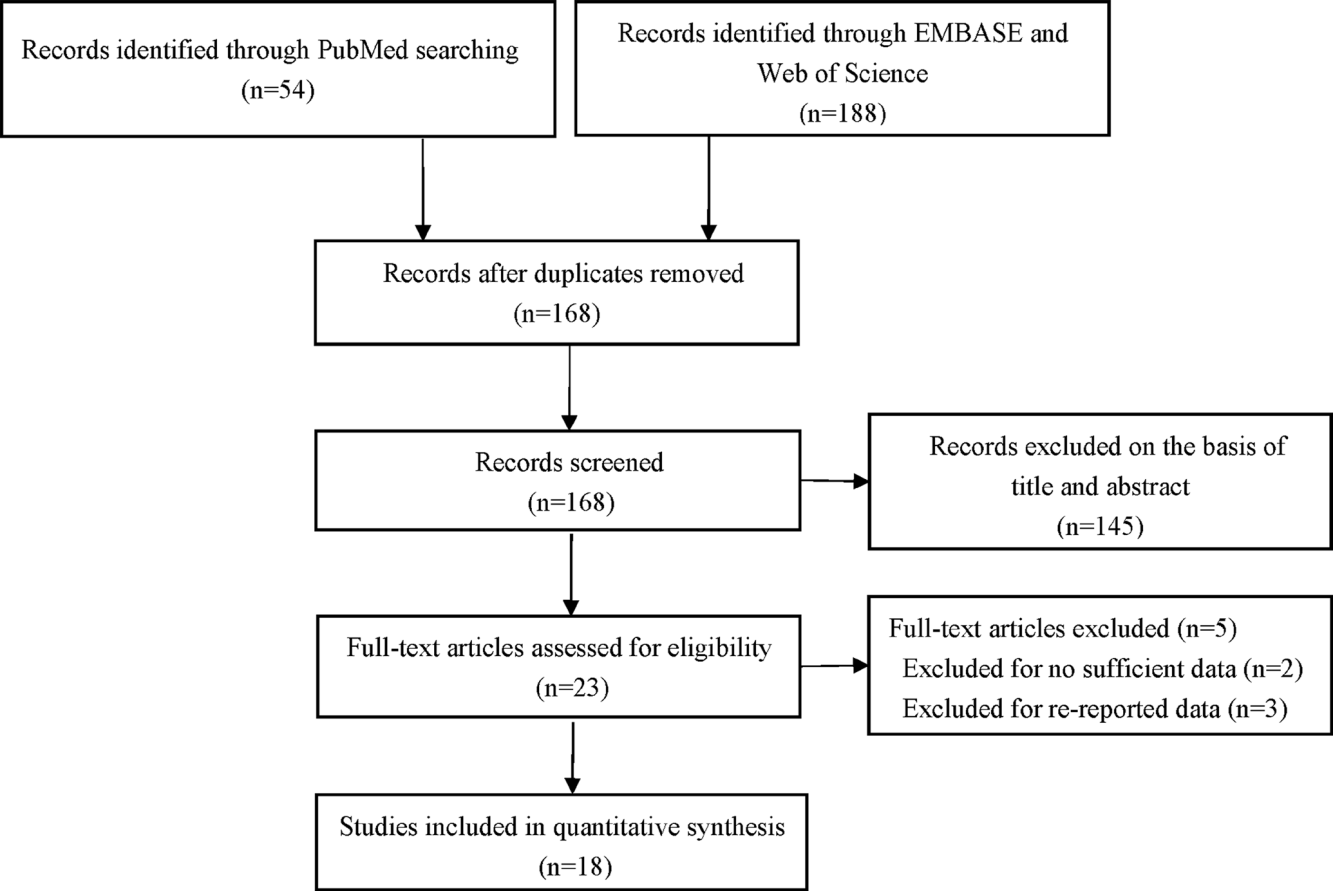
Flow diagram of the selection of the studies in this meta-analysis

The complete search process is presented in Figure 1. A total of 242 publications were preliminarily identified according to the search strategy described in the methods and materials section. After removing duplicate articles, 168 records remained. Then we read titles and abstracts of all the studies and excluded 145 articles that were obviously unrelated. After carefully reviewing the full texts of the remaining articles, an additional five articles were excluded, including two articles that had no sufficient data and three articles that contained re-reported data. Ultimately, thirty-eight eligible studies from eighteen remaining articles [9–26], including 17,417 cases and 18,988 cancer-free controls, were eventually included in our meta-analysis. The characteristics of the thirty-eight eligible studies are presented in Table 1. Among these included studies, twenty-five were performed in Asians [9, 11–16, 20–22], twelve in Caucasians [10, 17–18, 23–26], and one in mixed ethnicity [19]. All the cases in the included studies were in accordance with the pathological diagnostic criteria of breast cancer and all the papers were published between 2009 and 2016.

**Table 1:** Characteristics of studies included in the meta-analysis

First author	Year	Country	Ethnicity	Genotyping method	Number (case/control)	HWE (*P* value)
rs3746444						
Hu[9]	2009	China	Asian	PCR-RFLP	1009/1093	0.057
Catucci[10]	2010	Italy	Caucasian	Taqman	756/1242	0.250
Catucci[10]	2010	Germany	Caucasian	Taqman	823/925	0.893
Alshatwi[11]	2012	Saudi	Asian	Taqman	100/100	0.227
Bansal[12]	2014	India	Asian	PCR-RFLP	121/164	0.261
Omrani[13]	2014	Iran	Asian	TARMS-PCR	236/203	0.241
Qi[14]	2015	China	Asian	Taqman	321/290	0.053
He[15]	2015	China	Asian	MassARRAY	450/450	0.143
Dai[16]	2016	China	Asian	MassARRAY	560/583	0.131
rs11614913						
Hu[9]	2009	China	Asian	PCR-RFLP	1009/1093	0.210
Hoffman[17]	2009	USA	Caucasian	MassARRAY	426/466	0.583
Catucci[10]	2010	Italy	Caucasian	Taqman	751/1243	0.315
Catucci[10]	2010	Germany	Caucasian	Taqman	1101/1496	0.711
Jedlinski[18]	2011	Australia	Caucasian	PCR-RFLP	187/171	0.830
Alshatwi[11]	2012	Saudi	Asian	Taqman	100/100	0.032
Linhares[19]	2012	Brazil	Mixed	Taqman	388/388	0.005
Zhang[20]	2012	China	Asian	PCR-RFLP	248/243	0.893
Ma[21]	2013	China	Asian	MassARRAY	190/187	0.037
Bansal[12]	2014	India	Asian	PCR-RFLP	121/165	0.042
Omrani[13]	2014	Iran	Asian	TARMS-PCR	236/203	0.000
Qi[14]	2015	China	Asian	Taqman	321/290	0.141
He[15]	2015	China	Asian	MassARRAY	450/450	0.990
Zhang[22]	2015	China	Asian	MassARRAY	379/187	0.037
Dai[16]	2016	China	Asian	MassARRAY	560/583	0.540
Morales[23]	2016	Chile	Caucasian	Taqman	440/807	0.121
rs2910164						
Hu[13]	2009	China	Asian	PCR-RFLP	1009/1093	0.221
Catucci[10]	2010	Germany	Caucasian	Taqman	805/904	0.753
Catucci[10]	2010	Italy	Caucasian	Taqman	754/1243	0.019
Pastrello[24]	2010	Italy	Caucasian	Sequencing	88/155	0.332
Garcia[25]	2011	France	Caucasian	Taqman	1130/596	0.150
Alshatwi[11]	2012	Saudi	Asian	Taqman	100/100	0.051
Ma[21]	2013	China	Asian	MassARRAY	192/191	0.983
Bansal[12]	2014	India	Asian	PCR-RFLP	121/164	0.130
Omrani[13]	2014	Iran	Asian	TARMS-PCR	236/203	0.000
Qi[14]	2015	China	Asian	Taqman	321/290	0.013
He[15]	2015	China	Asian	MassARRAY	450/490	0.478
Zhang[22]	2015	China	Asian	MassARRAY	382/191	0.983
Upadhyaya[26]	2015	Australia	Caucasian	HRM	546/246	0.091

Abbreviations: HWE: Hardy-Weinberg equilibrium for controls. PCR-RFLP: polymerase chain reaction-restriction fragment length polymorphism. TARMS-PCR: tetra-primer amplification refractory mutation system-polymerase chain reaction. HRM: High ResolutionMelting.

### Meta-analysis results

**Figure 2:**
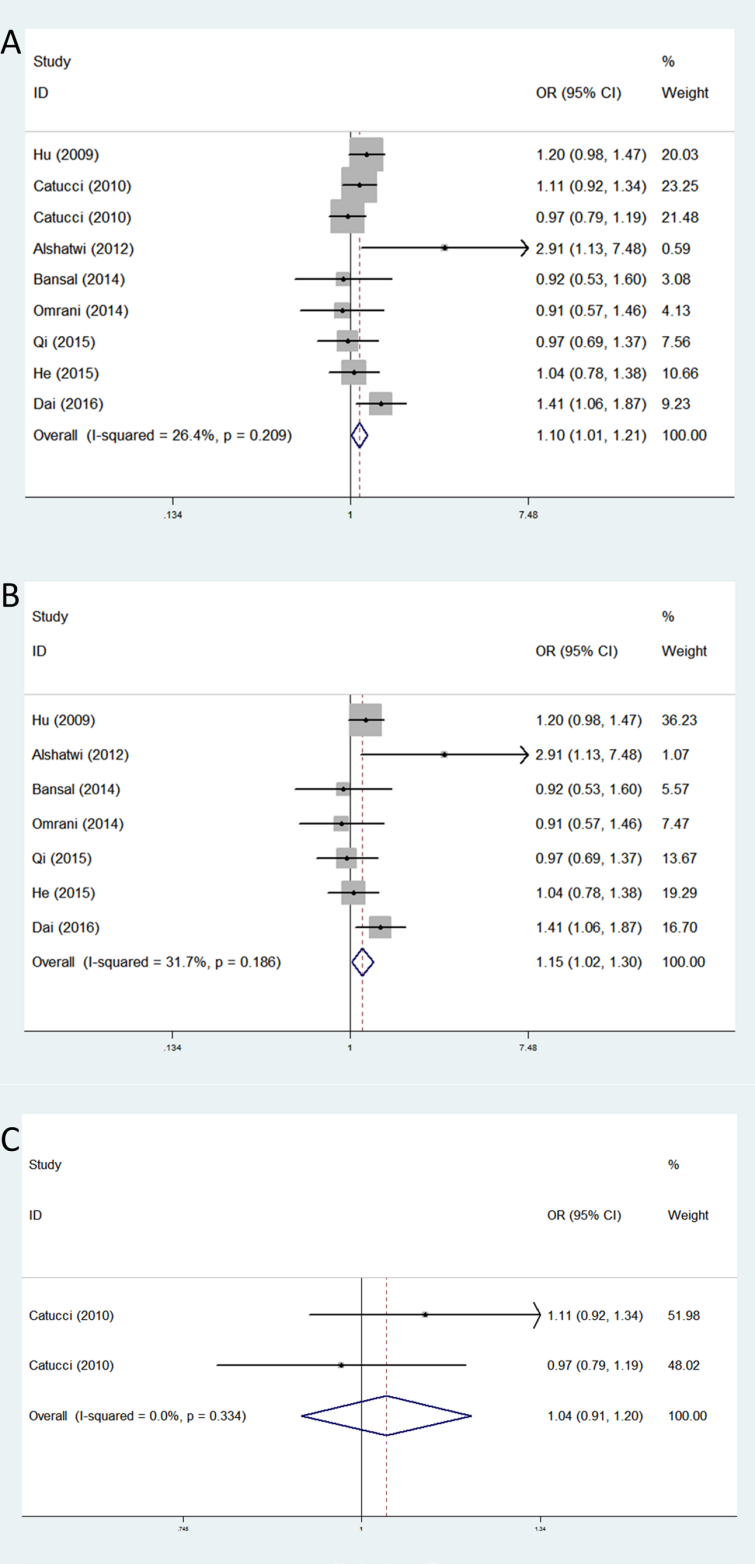
Forest plots of associations between rs3746444 and breast cancer risk among different ethnic groups in heterozygote genetic model (**A**) the overall populations; (**B**) Asians; (**C**) Caucasians.

Distribution and allele frequency of these three microRNA polymorphisms in cases and controls are shown in Table 2, and the main results of this meta-analysis are presented in Table 3. For rs3746444, the nine eligible studies with 4,376 breast cancer patients and 5,050 cancer-free controls were finally included. As shown in Table 3, we observed an increased breast cancer risk associated with rs3746444 polymorphism in the four genetic models: allele contrast genetic model (OR = 1.17, 95% CI = 1.04–1.31, *P* = 0.008), homozygote genetic model (OR = 1.41, 95% CI = 1.19–1.67, *P* < 0.001), heterozygote genetic model (OR = 1.10, 95% CI = 1.01–1.21, *P* = 0.036), and dominant genetic model (OR = 1.16, 95% CI = 1.06–1.26, *P* = 0.001). The stratified analysis by ethnicity showed an increased BC risk in Asians (allele contrast genetic model: OR = 1.08, 95% CI = 1.03–1.14, *P* = 0.001; homozygote genetic model: OR = 1.11, 95% CI = 1.03–1.21, *P* = 0.009; heterozygote genetic model: OR = 1.11, 95% CI = 1.03–1.20, *P* = 0.008; dominant genetic model: OR = 1.17, 95% CI = 1.07–1.29, *P* = 0.001). However, no meaningful correlation was observed in Caucasians (Table 3) (Figure 2).

**Table 2:** Genotype distribution and allele frequency of these three microRNA polymorphisms (miR-499 rs3746444, miR-196a rs11614913 and miR-146a rs2910164) in cases and controls

First author
	Genotype (N)						Allele frequency (N)					
	Case				Control				Case		Control	
	Total	AA	AB	BB	Total	AA	AB	BB	A	B	A	B
miR-499 rs3746444												
Hu	1009	707	258	44	1093	816	248	29	1672	346	1880	306
Catucci	756	414	295	47	1242	704	452	86	1123	389	1860	624
Catucci	823	536	250	37	925	601	290	34	1322	324	1492	358
Alshatwi	100	30	62	8	100	45	40	15	78	122	70	130
Bansal	121	80	30	11	164	106	43	15	190	52	255	73
Omrani	236	131	44	61	203	130	48	25	306	166	308	98
Qi	321	152	117	52	290	141	112	37	421	221	394	186
He	450	184	177	89	450	203	188	59	545	355	594	306
Dai	560	407	135	18	583	463	109	11	949	171	1035	131
miR-196a rs11614913												
Hu	1009	287	483	239	1093	358	517	218	1057	961	1233	953
Hoffman	426	181	209	36	466	166	229	71	571	281	561	371
Catucci	751	334	330	87	1243	532	550	161	998	504	1614	872
Catucci	1101	432	512	157	1496	584	696	216	1376	826	1864	1128
Jedlinski	187	68	86	33	171	58	82	31	222	152	198	144
Alshatwi	100	35	63	2	100	46	50	4	133	67	142	58
Linhares	388	94	177	117	388	127	165	96	365	411	419	357
Zhang	248	148	89	11	243	133	93	17	385	111	359	127
Ma	190	54	92	44	187	59	79	49	200	180	197	177
Bansal	121	68	41	12	165	85	59	21	177	65	229	101
Omrani	236	218	18	0	203	178	25	0	454	18	381	25
Qi	321	168	119	34	290	185	88	17	455	187	458	122
He	450	81	233	136	450	93	223	134	395	505	409	491
Zhang	379	108	181	90	187	59	79	49	397	361	197	177
Dai	560	197	265	98	583	155	284	144	659	461	594	572
Morales	440	192	191	57	807	342	351	114	575	305	1035	579
miR-146a rs2910164												
Hu	1009	329	515	165	1093	362	551	180	1173	845	1275	911
Catucci	805	451	304	50	904	536	318	50	1206	404	1390	418
Catucci	754	409	286	59	1243	650	520	73	1104	404	1820	666
Pastrello	88	53	30	5	155	90	59	6	136	40	239	71
Garcia	1130	676	388	66	596	352	220	24	1740	520	924	268
Alshatwi	100	48	50	2	100	51	46	3	146	54	148	52
Ma	192	63	94	35	191	64	93	34	220	164	221	161
Bansal	121	82	35	4	164	84	72	8	199	43	240	88
Omrani	236	183	45	8	203	155	39	9	411	61	349	57
Qi	321	146	132	43	290	126	144	20	424	218	396	184
He	450	75	242	133	490	112	225	153	392	508	449	531
Zhang	382	126	181	75	191	64	93	34	433	331	221	161
Upadhyaya	546	325	193	28	246	112	99	35	843	249	323	169

A: the major allele; B: the minor allele.

**Table 3:** Meta-analysis results

Comparisons	OR	95%CI	P(OR) I^2^	Heterogeneity		Effects model	P(Begg)	P(Egger)
				*P*				
Allele contrast genetic model								
rs3746444	1.17	1.04–1.31	0.008	58%	0.014	R	0.602	0.796
Asian	1.24	1.08–1.42	0.002	48%	0.071	R	-	-
Caucasian	1.03	0.92–1.15	0.628	0%	0.924	F	-	-
rs11614913	0.98	0.89–1.09	0.748	73%	0.000	R	0.753	0.718
Asian	0.99	0.85–1.17	0.932	76%	0.000	R	-	-
Caucasian	0.93	0.87–1.00	0.044	37%	0.173	F	-	-
rs2910164	0.97	0.87–1.07	0.510	64%	0.001	R	0.161	0.292
Asian	1.02	0.94–1.10	0.702	16%	0.303	F	-	-
Caucasian	0.92	0.74–1.14	0.453	84%	0.000	R	-	-
Homozygote genetic model								
rs3746444	1.41	1.19–1.67	0.000	34%	0.147	F	1.000	0.768
Asian	1.64	1.34–2.02	0.000	0%	0.543	F	-	-
Caucasian	1.03	0.77–1.38	0.844	0%	0.381	F	-	-
rs11614913	0.95	0.77–1.16	0.600	71%	0.000	R	0.488	0.538
Asian	0.98	0.71–1.34	0.877	73%	0.000	R	-	-
Caucasian	0.85	0.73–0.99	0.037	52%	0.080	R	-	-
rs2910164	1.03	0.80–1.33	0.804	64%	0.001	R	0.246	0.554
Asian	1.11	0.94–1.32	0.221	0%	0.474	F	-	-
Caucasian	0.95	0.52–1.72	0.862	85%	0.000	R	-	-
Heterozygote genetic model								
rs3746444	1.10	1.01–1.21	0.036	26%	0.209	F	1.000	0.610
Asian	1.15	1.02–1.30	0.022	32%	0.186	F	-	-
Caucasian	1.04	0.91–1.20	0.572	0%	0.334	F	-	-
rs11614913	1.03	0.92–1.15	0.577	47%	0.019	R	0.964	0.671
Asian	1.07	0.89–1.28	0.481	57%	0.014	R	-	-
Caucasian	0.95	0.86–1.06	0.348	0%	0.887	F	-	-
rs2910164	0.95	0.83–1.08	0.403	54%	0.011	R	0.951	0.598
Asian	0.98	0.80–1.20	0.866	58%	0.019	R	-	-
Caucasian	0.91	0.77–1.07	0.241	51%	0.085	R	-	-
Dominant genetic model								
rs3746444	1.16	1.06–1.26	0.001	19%	0.272	F	0.466	0.332
Asian	1.25	1.12–1.40	0.000	0%	0.509	F	-	-
Caucasian	1.04	0.91–1.89	0.569	0%	0.536	F	-	-
rs11614913	1.01	0.89–1.15	0.837	66%	0.000	R	0.893	0.885
Asian	1.08	0.89–1.32	0.442	72%	0.000	R	-	-
Caucasian	0.93	0.84–1.02	0.136	0%	0.533	F	-	-
rs2910164	0.95	0.83–1.08	0.453	59%	0.003	R	0.855	0.478
Asian	1.00	0.83–1.19	0.960	50%	0.053	R	-	-
Caucasian	0.90	0.73–1.11	0.317	73%	0.005	R	-	-
Recessive genetic model								
rs3746444	1.29	0.97–1.71	0.083	66%	0.003	R	0.754	0.883
Asian	1.38	0.97–1.96	0.070	67%	0.006	R	-	-
Caucasian	1.01	0.75–1.34	0.971	11%	0.289	F	-	-
rs11614913	0.93	0.80–1.08	0.324	58%	0.002	R	0.138	0.286
Asian	0.98	0.79–1.21	0.843	62%	0.005	R	-	-
Caucasian	0.88	0.76–1.01	0.062	45%	0.120	F	-	-
rs2910164	1.03	0.82–1.29	0.784	62%	0.001	R	0.669	0.879
Asian	1.03	0.89–1.19	0.686	14%	0.319	F	-	-
Caucasian	1.00	0.57–1.73	0.986	83%	0.000	R	-	-

F: fixed effects model; R: random effects model.

**Figure 3:**
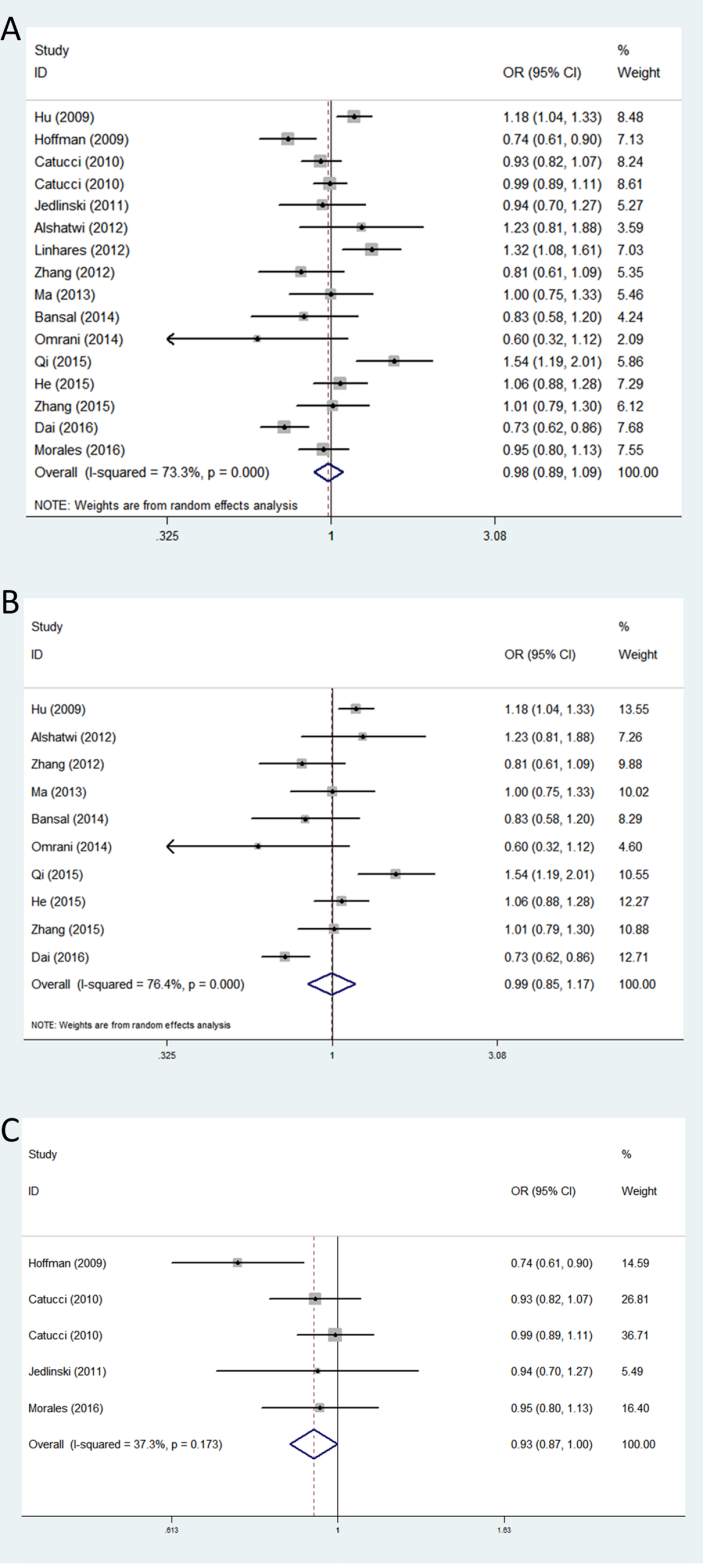
Forest plots of associations between rs11614913 and breast cancer risk among different ethnic groups in allele contrast genetic model (**A**) the overall populations; (**B**) Asians; (**C**) Caucasians.

For rs11614913, the association of this SNP with breast cancer risk was investigated in sixteen studies involving 6,907 cases and 8,072 control subjects. We failed to find a significant association between this polymorphism and BC risk in any of the five genetic models in the overall populations. However, in the subgroup analysis by ethnicity, we found rs11614913 was associated with a decreased risk of breast cancer among Caucasians in allele contrast genetic model (OR = 0.93, 95% CI = 0.87–1.00, *P* = 0.044) (Table 3) (Figure 3).

**Figure 4:**
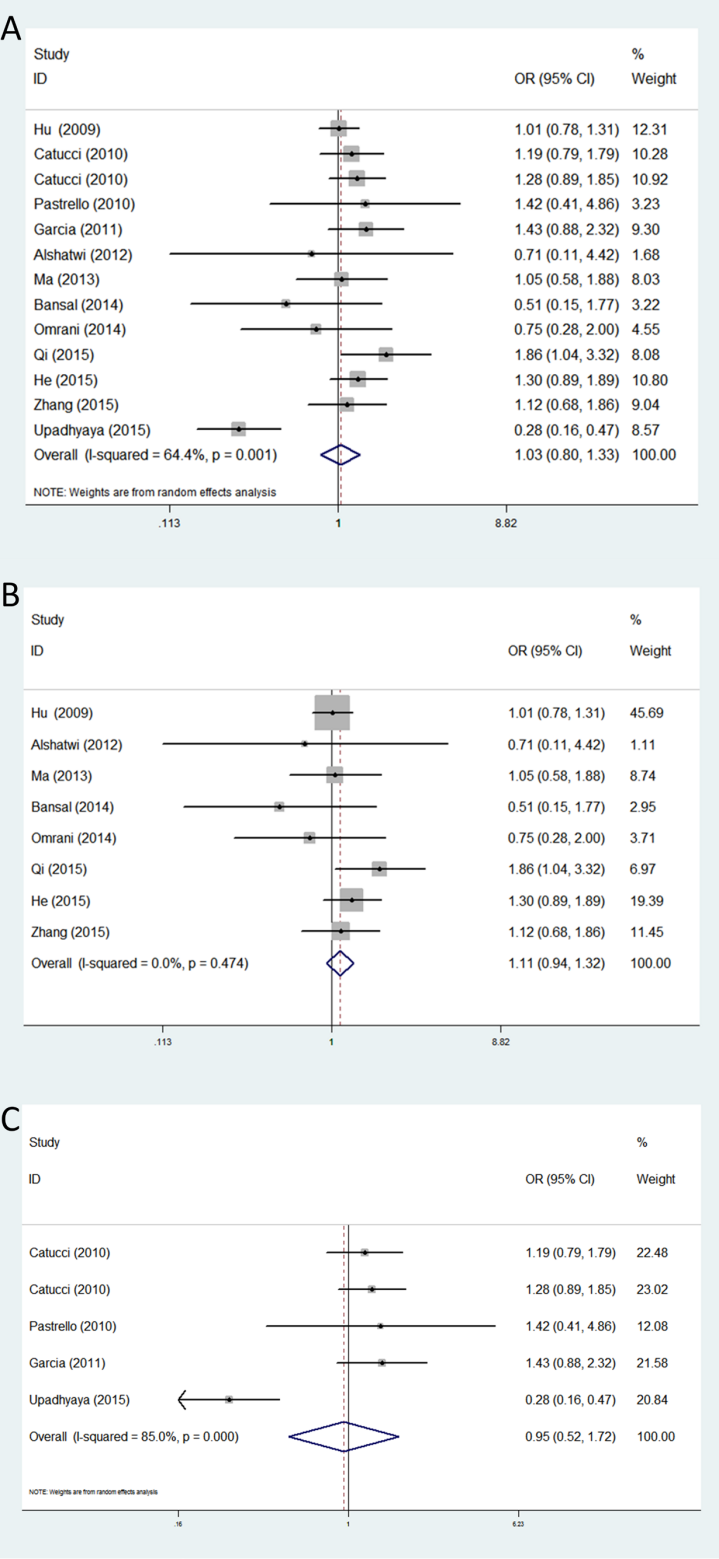
Forest plots of associations between rs2910164 and breast cancer risk among different ethnic groups in homozygote genetic model (**A**) the overall populations; (**B**) Asians; (**C**) Caucasians.

For rs2910164, thirteen studies with 6,134 cases and 5,866 controls were used to assess the association between this genetic polymorphism and breast cancer susceptibility. No obvious association was found between the rs2910164 polymorphism and breast cancer risk in any of the five genetic models. Similarly, further stratified analysis by ethnicity showed no significant correlation between rs11614913 and breast cancer susceptibility in all the ethnic groups (Table 3) (Figure 4).

### Sensitivity analysis

**Figure 5:**
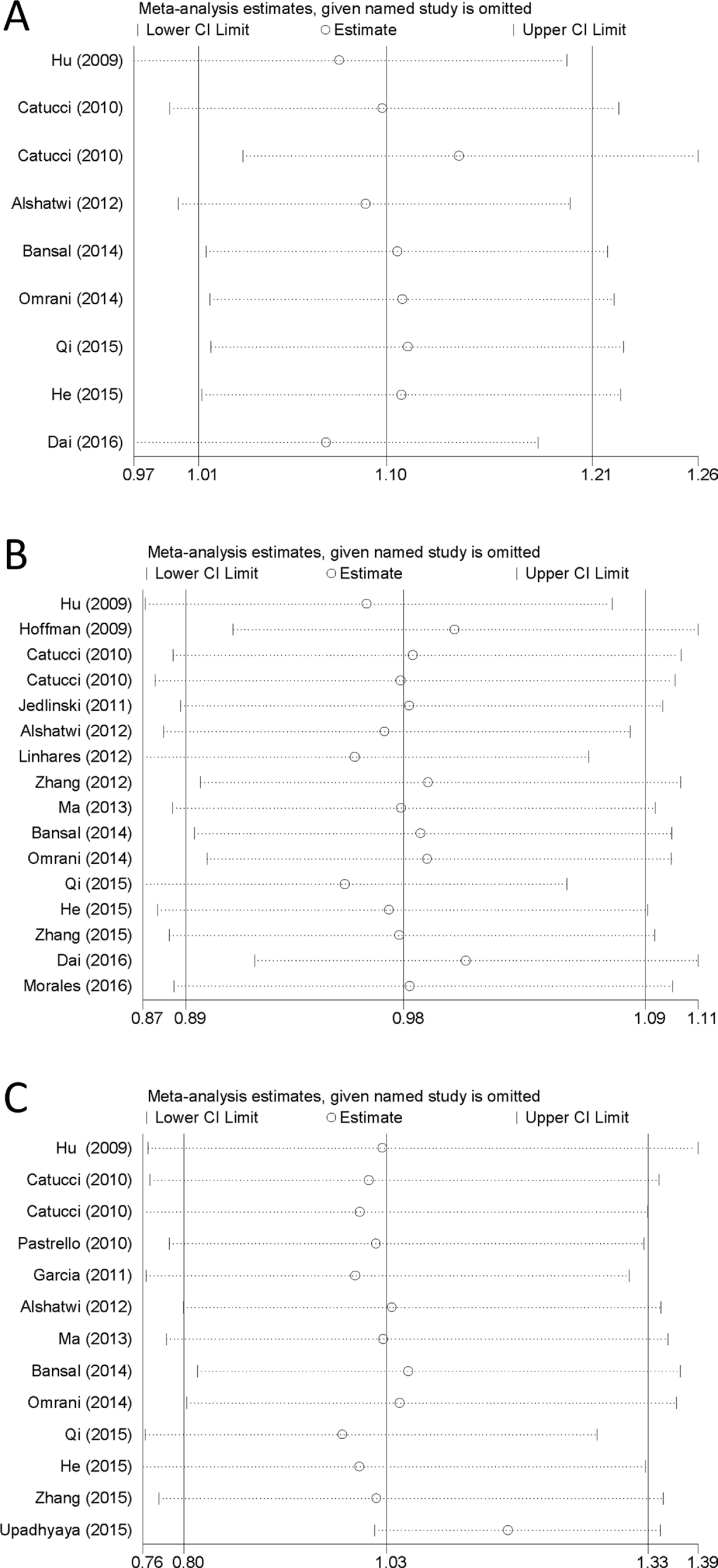
Sensitivity analyses of the three microRNA polymorphisms in specific genetic models (**A**) rs3746444 in heterozygote genetic model; (**B**) rs11614913 in allele contrast genetic model; (**C**) rs2910164 in homozygote genetic model.

In all the included studies, nine studies were not consistent with the Hardy-Weinberg equilibrium (HWE) in controls (*P* < 0.05) (Table 1). Nevertheless, after conducting the sensitivity analyses, the pooled ORs were no statistically significant change when deleting any of the studies, demonstrating that our results are stable and reliable (Figure 5).

### Heterogeneity analysis

We used Q statistic to determine the heterogeneity among studies in this meta-analysis. If significant heterogeneity existed (*P* value of *Q* test was < 0.1), we would select random-effects model to perform related statistical analysis; if not, we would choose fixed-effects model to carry out our research.

### Publication bias

**Figure 6:**
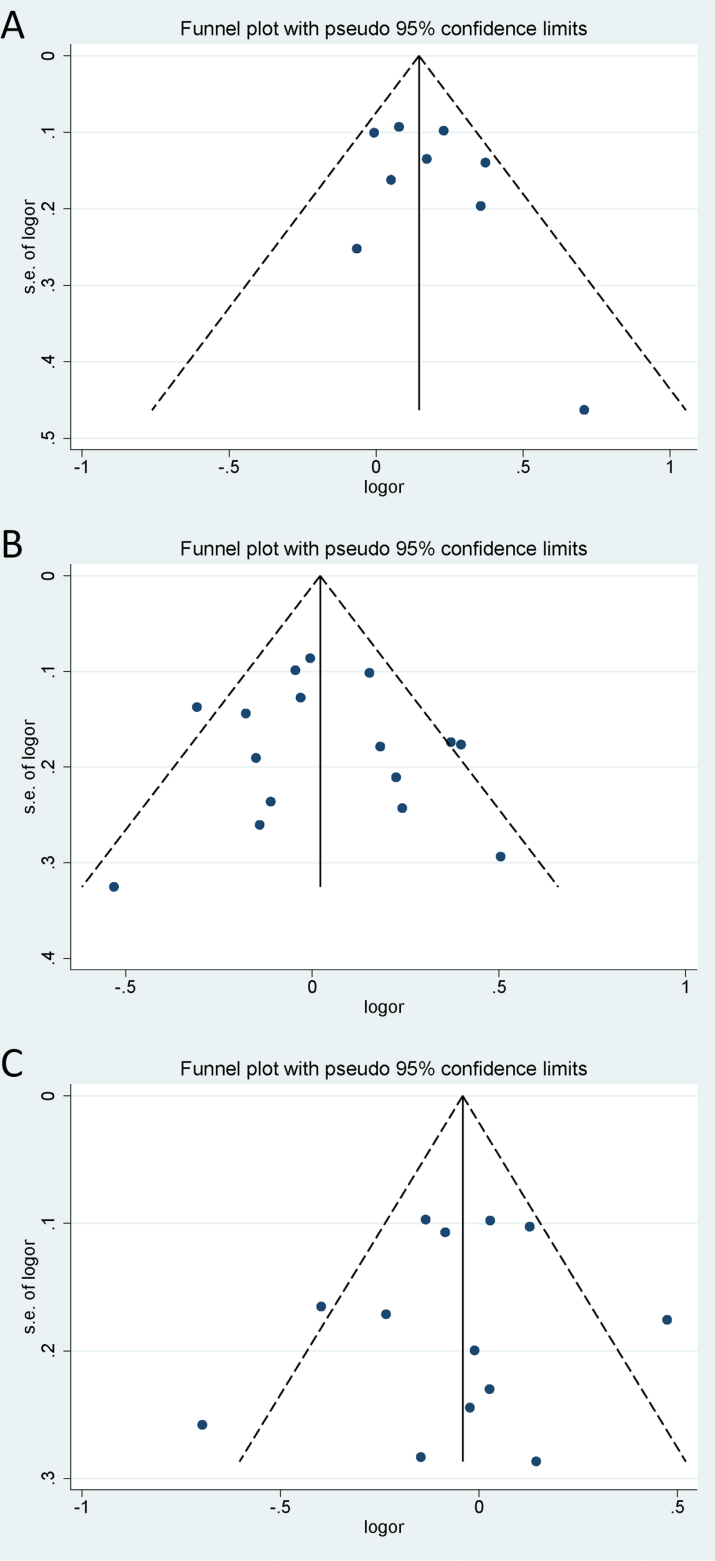
Funnel plots of the three microRNA polymorphisms in specific genetic models (**A**) rs3746444 in dominant genetic model; (**B**) rs11614913 in heterozygote genetic model; (**C**) rs2910164 in heterozygote genetic model.

**Figure 7:**
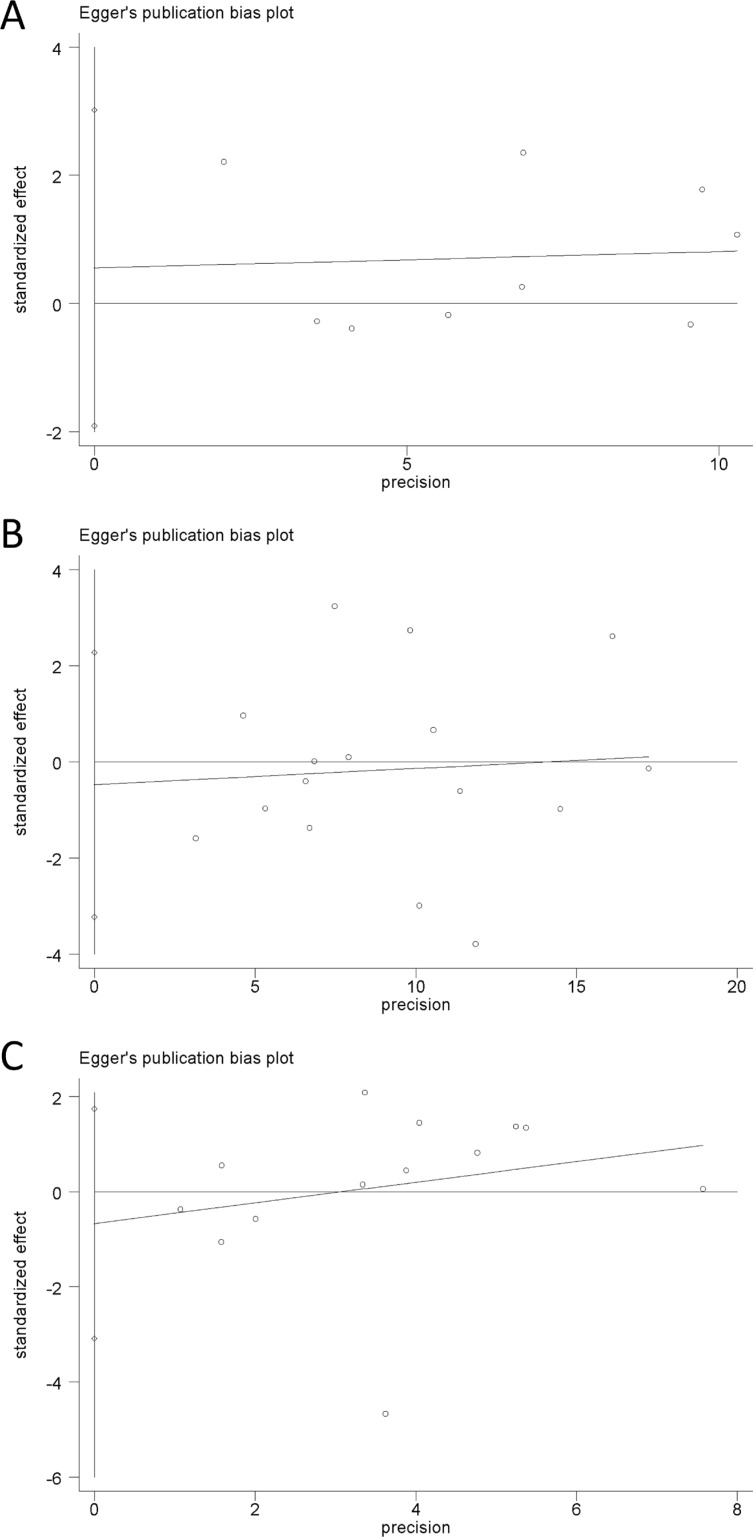
Egger's publication bias plots of the three microRNA polymorphisms in specific genetic models (**A**) rs3746444 in heterozygote genetic model; (**B**) rs11614913 in allele contrast genetic model; (**C**) rs2910164 in homozygote genetic model.

Begg's test, Egger's test and funnel plot were all used to assess the publication bias of the included studies. All *P* values of Begg's test and Egger's test were greater than 0.05 (*P* > 0.05), demonstrating that there is no significant publication bias in the overall population (Table 3). Funnel plot also proved that publication bias did not exist with no obvious asymmetry that could be observed (Figure 6). Hence, no publication bias was found in this meta-analysis. Egger's publication bias plots are shown in Figure 7.

## DISCUSSION

With the development of science and the improvement of medical technology, the diagnosis and treatment of breast cancer has made great progress in the past years. However, its pathogenesis has not been completely elucidated yet. Breast cancer is a highly heterogeneous disease. Its occurrence and development involves oncogene activation, tumor suppressor gene inactivation and many other related factors. In recent years, many microRNA polymorphisms have been identified as risk factors for breast cancer [29, 30].

Currently, three well-known SNPs in microRNA (rs3746444, rs11614913 and rs2910164) have been widely investigated and found to be associated with the risk of several types of cancer [31–33]. Nevertheless, the relationship between these three miRNA polymorphisms and BC risk can't be determined because of inconsistent results published articles reported. Consequently, in order to obtain a more precise evaluation of the relationship, we perform this meta-analysis.

The microRNA-499 rs3746444 polymorphism is located in chromosome 20q11.22, which is an A to G single-nucleotide mutation that occurs in the stem structure of miR-499 precursor [34]. Studies have shown that rs3746444 can regulate the expression of SOX genes [35]. The abnormal expression of SOX genes can activate Wnt/β-catenin signaling pathway, which is associated with breast tumorigenesis and progression, so rs3746444 may play an important role in the occurrence and development of breast cancer by altering SOX genes' expression level. Several studies [9, 13, 15–16] reported that rs3746444 polymorphism had an increased association with breast cancer risk, while others [10, 27, 36] showed no significant association between rs3746444 and BC susceptibility. Our result indicated that SNP rs3746444 was associated with BC risk in the four genetic models except recessive genetic model. In the subgroup analysis by ethnicity, we found that rs3746444 was associated with an increased risk of BC in Asians; nevertheless, no significant association was observed in Caucasians. The result was in correspondence with that of two previously published meta-analyses [37, 38], which further demonstrates that our result is credible.

Genetic variant in miR-196a2 (rs11614913) involving a C to T nucleotide substitution can alter its expression and function, which is associated with cancer susceptibility. Studies have reported that miR-196a can repress HOX gene expression through directing its mRNA cleavage [39]. Recent studies have found that HOXBP is overexpressed in breast cancer and it can promote invasion and metastasis of breast cancer [40]. Besides, the study by Seki et al. demonstrated that HOXBP was a significant prognostic factor in BC [41]. However, published studies showed inconsistent results on the association between rs11614913 and BC risk. Dai et al. [16] reported that rs11614913 polymorphism was a protective factor of BC. On the contrary, rs1614913 was found to be associated with an increased risk of BC in other studies [9, 14]. In our study, no correlation was detected between this polymorphism and breast susceptibility in the overall populations. However, in the subgroup analysis by ethnicity, we observed that rs11614913 was associated with a decreased risk of breast cancer among Caucasians in allele contrast genetic model. Our finding was partly consistent with the results of three previously published meta-analyses: in the meta-analysis by Chen et al. [42], twelve studies were included and the result showed that rs11614913 was a protective factor of BC in Asians; in other two meta-analyses [27–28], ten studies and eight studies were included, respectively, demonstrating that the rs11614913 polymorphism could decrease the BC risk in the overall populations. Compared with them, our study included eighteen eligible studies so our result was more reliable with the larger sample size. But consider the obvious heterogeneity among the included studies, we should cautiously treat our result although sensitivity analysis demonstrated that our result was stable.

For rs2910164, we observed that there was no association between rs2910164 and breast cancer risk in the general populations. When stratified by ethnicity, similar results could be seen in both Asians and Caucasians. Nevertheless, in a previous meta-analysis by Dai et al. [28], the authors found that the rs2910164 polymorphism had a significant association with BC risk in Caucasians using the homozygote comparison model and the dominant model. This contradiction may be due to different sample sizes and racial groups of the two studies: compared with his study, our study includes five new case-control studies, which will expand the sample size and thus get a more precise evaluation of association between rs2910164 and BC risk.

Some limitations of this meta-analysis must be pointed out. First, several important individual information was not provided, thus we couldn't perform a more accurate analysis stratified by other risk factors of breast cancer such as age, gender, lifestyle and environmental factor. Meanwhile, a few studies selected specific type of breast cancer as the subjects of case group: the study by Ma et al. focused on triple negative breast cancer [21]; study by Catucci et al. only involved familial BC [10]. Second, some studies didn't conform to Hardy-Weinberg equilibrium (HWE) in controls, which might influence the reliability of the results. Third, the variety of genotyping methods used in the included studies might have an impact on the results of our study. Last, obvious between-study heterogeneity existed in the included studies, and its sources were not clear. Moreover, not sufficient studies also made it difficult to make a more accurate assessment of these three polymorphisms and breast cancer susceptibility.

In summary, this meta-analysis indicates that miR-499 rs3746444 is associated with an increased BC risk in Asians and in the overall populations, while miR-196a rs11614913 has a decreased association with breast cancer risk among Caucasians. Besides, miR-146a rs2910164 has no relationship with breast cancer susceptibility. More multicenter studies with larger sample sizes are needed to further confirm the possible roles of these three microRNA polymorphisms in breast cancer.

## MATERIALS AND METHODS

### Literature and search strategy

We searched PubMed, EMBASE and Web of Science databases for papers published before September 18, 2016. There were no language restrictions in our searching process. The searching strategy was as follow: (breast cancer OR breast carcinoma) AND (polymorphism OR variant OR genotype OR SNP) AND (miR-499 OR rs3746444 OR miR-196a OR rs11614913 OR miR-146a OR rs2910164). Besides, the references of the retrieved studies were also reviewed to find additional eligible publications.

### Inclusion criteria

All included studies must meet the following criteria: (1) evaluation of these three microRNA polymorphisms (miR-499 rs3746444, miR-196a rs11614913 and miR-146a rs2910164) and BC risk; (2) case-control studies; (3) sufficient genotyping data that could be used to calculate odds ratios (ORs) and 95% confidence intervals (CIs); (4) all the breast cancer subjects in case groups must be pathologically confirmed. The exclusion criteria were: (1) not case-control studies; (2) case reports, editorials, comments or review articles; (3) duplicate studies; (4) no detailed genotyping data.

### Data extraction

Two investigators independently extracted the data from the included studies, and discrepancies were resolved through discussion with a third researcher. The following information was extracted: the first author, year of publication, country of origin, ethnicity, genotyping method, number of cases and controls, and *P* value for Hardy-Weinberg equilibrium (HWE).

### Statistical analysis

The association of these three functional microRNA polymorphisms with BC susceptibility was measured by pooled odds ratios (ORs) and 95% confidence intervals (CIs) in five genetic models, including a allele contrast genetic model, a homozygote genetic model, a heterozygote genetic model, a dominant genetic model, and a recessive genetic model. Heterogeneity among studies was evaluated by *I*^2^ test and *Q* test. For *I*^2^ test, the criteria for heterogeneity were as follows: *I*^2^ < 25%, no heterogeneity; 25%-75%, moderate heterogeneity; *I*^2^ > 75%, high heterogeneity. If the *P value* of *Q* test was < 0.1, the random-effects model was used; otherwise, the fixed-effects model was applied. Sensitivity analysis was performed by sequentially deleting each study at a time to assess the influence of each study on the pooled ORs. We used Begg's test, Egger's test and funnel plot to assess publication bias. *P value* for Hardy-Weinberg equilibrium (HWE) was calculated by chi-square test in the control group of each study. Subgroup analysis was performed according to ethnicity. All statistical analyses were performed using STATA version 10.0 software (StataCorp LP, College Station, TX, USA). All *P* values were two sided, and *P* < 0.05 was considered statistically significant.
